# COPA Syndrome—From Pathogenesis to Treatment

**DOI:** 10.3390/diagnostics14242819

**Published:** 2024-12-14

**Authors:** Vlad Padureanu, Mircea-Cătălin Forțofoiu, Ionut Donoiu, Eugen-Nicolae Tieranu, Catalin Dumitrascu, Rodica Padureanu, Anca Emanuela Mușetescu, Cristina Alexandru, Carmen Catalina Iorgus, Florin Bobirca, Ana Dascalu, Anca Bobirca

**Affiliations:** 1 Department of Internal Medicine, University of Medicine and Pharmacy of Craiova, 200349 Craiova, Romania; vlad.padureanu@umfcv.ro (V.P.); catalin.fortofoiu@umfcv.ro (M.-C.F.); rodica.padureanu@umfcv.ro (R.P.); 2 Department of Cardiology, University of Medicine and Pharmacy of Craiova, 200349 Craiova, Romania; ionut.donoiu@umfcv.ro; 3 Department of Internal Medicine and Rheumatology, “Dr. Ion Cantacuzino” Clinical Hospital, 011437 Bucharest, Romania; maria-cristina.steopoaie@rez.umfcd.ro (C.A.); carmen-catalina.iorgus@rez.umfcd.ro (C.C.I.); anca.bobirca@umfcd.ro (A.B.); 4 Department of Rheumatology, University of Medicine and Pharmacy of Craiova, 200349 Craiova, Romania; anca.musetescu@umfcv.ro; 5 Department of Internal Medicine and Rheumatology, “Carol Davila” University of Medicine and Pharmacy, 050474 Bucharest, Romania; 6 Department of General Surgery, “Carol Davila” University of Medicine and Pharmacy, “Dr. Ion Cantacuzino” Clinical Hospital, 030167 Bucharest, Romania; florin.bobirca@umfcd.ro; 7 Department of Ophthalmology, Emergency University Hospital Bucharest, 050098 Bucharest, Romania; ana.dascalu@umfcd.ro

**Keywords:** COPA syndrome, diffuse alveolar hemorrhaging, interstitial lung disease, arthritis, immune dysregulation

## Abstract

Coatomer subunit α (COPA) syndrome is a mendelian autosomal dominant immune dysregulation disease characterized by early onset lung disease in the form of diffuse alveolar hemorrhaging or interstitial lung disease, frequently associated with arthritis, glomerulonephritis, and high titer autoantibodies usually mimicking other autoimmune diseases. While immunosuppressive medication has been effective in controlling arthritis, data on long-term lung disease control remains scarce, which poses a real challenge as the progression of lung disease is the main cause of poor life expectancy in COPA patients. Nevertheless, JAK inhibitor therapy seems to be the most promising therapeutic choice now.

## 1. Introduction

COPA syndrome, an inflammatory Mendelian disease, is caused by missense mutations in the coatomer subunit α (COPA) protein, which is a component of coat protein complex I (COPI) and, as such, participates in the retrograde Golgi-to-ER trafficking. In 2019, COPA syndrome was categorized as a non-inflammasome-associated condition by the International Union of Immunological Societies Expert Committee (IUIS) [1,2]. COPA syndrome is an uncommon condition that can be challenging to diagnose and treat considering it is a rare genetic autoinflammatory disease that can damage the kidneys, joints, and lungs. Stimulator of interferon genes (STING)-associated vasculopathy with onset in infancy (SAVI), another Type I interferonopathy, and COPA syndrome have a lot in common in terms of pathophysiology and symptoms—both mendelian disorders that cause dysregulation of STING activity, manifesting as diffuse alveolar hemorrhaging (DAH) and interstitial lung disease (ILD) [3].

COPA syndrome was first reported in 2015, and its prevalence may be higher than previously anticipated [4]. The syndrome appears to have varied penetrance and affects males and females at roughly the same rate, with an autosomal dominant inheritance pattern. The COPA condition does not seem to be racially predisposed as Caucasian, Asian, African American, and Icelandic-Nordic people have all been reported to experience it.

Laboratory findings are usually nonspecific, mimicking other autoimmune or autoinflammatory diseases. Autoantibodies are usually present in the form of antinuclear antibodies (ANA), antineutrophil cytoplasmic antibodies (ANCA), or anti-cyclic citrullinated peptides (anti-CCP) antibodies/rheumatoid factor (RF), usually accompanied by biologic inflammatory syndrome with an elevated erythrocyte sedimentation rate (ESR) and/or C-reactive protein [5].

Pulmonary involvement is almost universal, usually presenting as ILD or DAH. Joint involvement is the most common extra-pulmonary manifestation, usually presenting as non-erosive arthritis. Renal involvement in the form of glomerulonephritis is characteristic, although less frequent than the other two primary manifestations [6].

Different patterns of lung inflammation and fibrosis are characteristics of the diverse group of illnesses known as ILD. Lung volume restriction and decreased diffusion capacity are characteristics of ILD. Depending on the kind of ILD, the restrictive process may be caused by fibrosis, which is primarily permanent, or acute and subacute inflammatory alterations, which may be reversible. The loss of functioning capillaries due to fibrosis, emphysema, or pulmonary hypertension can cause impaired gas exchange, thus leading to breathlessness, going as far as respiratory failure and eventually death [7].

Given that COPA is a condition that can mimic other autoimmune/autoinflammatory diseases—ANCA autoantibodies and lung hemorrhaging or glomerulonephritis are suggestive of ANCA vasculitis, ANA autoantibodies and joint/lung/kidney involvement is suggestive of systemic lupus erythematosus (SLE), and antiCCP antibodies/RF with arthritis is suggestive of rheumatoid arthritis (RA)—early diagnosis can be difficult to ascertain [6], especially considering that the ILD type of lung involvement can be present in plenty of other autoimmune diseases, including RA [7,8,9].

Considering that disease severity can vary from mild to life-threatening and exacerbations can be fatal, it is of utmost importance to know when to suspect COPA syndrome as genetic testing is not always available. When it comes to exacerbations involving the lung or kidney, immunosuppressive induction therapy and supportive therapy are required, even necessitating lung transplantation in some severe cases.

In an attempt to clarify COPA syndrome as an entity, its pathophysiology, clinical presentation, organ manifestations, diagnostic red flags, and therapeutic options will all be covered in this narrative review.

## 2. Materials and Methods

Applying the search terms “pathophysiology”, “clinical manifestations”, “treatment”, “therapeutic approach” “clinical presentations”, and “COPA syndrome,” articles were examined using the PubMed (National Institutes of Health, Bethesda, MD, USA), Scopus (Elsevier, Amsterdam, Netherlands), Google Scholar (Google, Mountain View, CA, USA), and Semantic Scholar (Allen Institute for AI, Seattle, WA, USA) databases. During the research, letters, remarks, and opinions were not taken into consideration, and only the most relevant articles were included in order to realize a comprehensive narrative review of COPA syndrome clinical manifestations and treatment.

One article reporting 2 cases of COPA syndrome with Behcet-like symptoms was not included due to considerable deviation from the regular COPA syndrome clinical presentation and the absence of lung involvement. One conference abstract detailing a cohort of lung transplant patients was included due to its high relevance to the subject matter.

## 3. Results

### 3.1. Pathophysiology

Since 2019—including in the most recent update in 2022 [10]—the expert committee on Inborn Errors of Immunity of the International Union of Immunological Societies (IUIS) has classified COPA syndrome as “auto-inflammatory, other.” However, since both the adaptive [11] and innate [12] immune elements are abnormal, it would be more accurate to refer to it as a syndrome of dysregulated immunity.

In eukaryotic cells, DNA is typically found in the nucleus and mitochondria. Nonetheless, there are some situations where DNA appears in the cytosol. Self-DNA that leaks from the nucleus or mitochondria and non-self-DNA from DNA viruses are the two main sources of cytosolic DNA. The host immunological response is triggered by cytosolic DNA. This immunological response appears to be largely dependent on the stimulator of interferon genes (STING) and cyclic GMP-AMP synthase [13].

STING, also known as MITA, ERIS, MPYS, or TMEM173, is an endoplasmic reticulum (ER) protein that plays a role in sensing cytosolic DNA or cyclic dinucleotides (CDNs), including cGAMP [13,14]. Following its binding to cGAMP, STING leaves the ER and moves to the Golgi, where it activates nuclear factor-kappa B (NF-κB) and recruits TANK-binding kinase-1 (TBK1), which in turn phosphorylates interferon regulatory factor 3 (IRF3) to cause type I interferon and proinflammatory responses. It is worth mentioning that STING also plays a role in other physiological processes such as cell death, autophagy, and senescence [13].

Anterograde traffic from the ER to the Golgi is facilitated by the coat protein complex II (COP-II), which is responsible for creating the membrane vesicles (COP-II vesicles) that sprout from the ER [15].

Notably, the examination of the Golgi using immunofluorescence microscopy indicates that the trans-Golgi network (TGN), a Golgi region in charge of sorting exocytic cargo molecules for delivery to the plasma membrane and endosomes, is the only location where STING recruitment of TBK1 is activated [16].

According to a number of studies, STING activation occurs in the ER-Golgi intermediate compartment (ERGIC), an intricate membrane system located between the ER, and the Golgi and is conventionally considered an ERGIC-53 (p58)-positive compartment. However, as ERGIC-53 moves between the ER, ERGIC, and the Golgi, caution must be used when interpreting STING and ERGIC-53 co-localization results. As previously stated, pTBK1, the active form of TBK1, only localizes at the TGN and not at the other Golgi domains, refuting the idea that STING activation occurs at the ERGIC [13,17].

**Figure 1:**
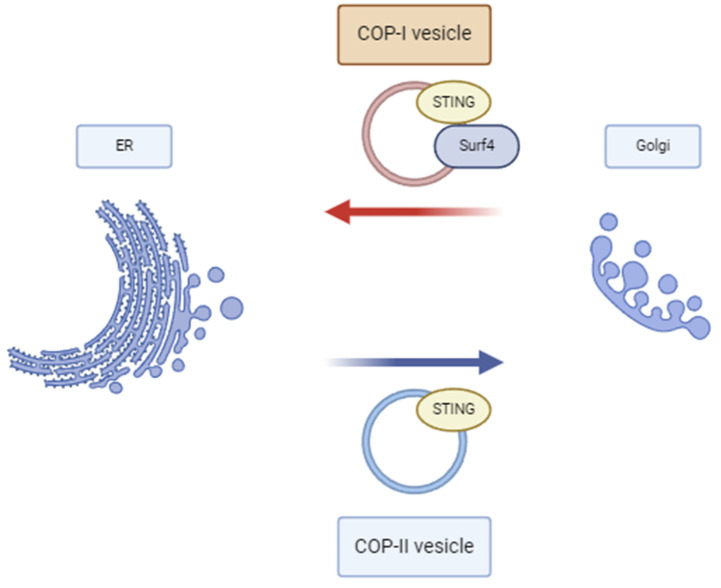
Anterograde and retrograde transport of STING.

The retrograde membrane transport from the Golgi to the ER is mediated by the COP-I complex by way of COP-I vesicles [17]. Notably, COP-I does not bind STING directly, instead forming a complex with the transport protein Surf4 [18]. STING transport between the ER and the Golgi has been illustrated in Figure 1.

The exact mechanism of how the impaired retrograde transport caused the typical manifestations of COPA syndrome remained largely unknown until relatively recently. Recent research shows that constitutive activation of STING, even in the absence of a proper ligand, along with a loss of STING’s ER localization, is the likely etiology of COPA syndrome [19]. Due to reduced COP-I transport caused by the disease-causing COP-α mutations, STING cannot be recovered from the Golgi and returned to the ER, thus leading to continuous activation of proinflammatory responses [12,13].

ER stress and continuous activation of the IFN I pathway leads to altered T cell thymic selection, skewing T cells toward the Th17 phenotype. The expression of Th17-related stimulant cytokines, such as IL-1β, IL-6, IL-17A, and IL-23, is also elevated by this rise in Th17 cells [11,20]. In their investigation on the subject, Zimu Deng et al. [11] came to an unexpected conclusion: STING plays a functional role in thymic stromal tissue. However, the processes controlling interferon production in the thymus are still mostly unknown, even though thymic epithelial cells are recognized as a major source of type I interferons [12].

In contrast to STING-induced IRF3 activation, NF-κB signaling remains intact when ER-to-Golgi STING transport is disrupted. Less is known about the exact process by which STING triggers NF-κB signaling. One suggestion is that ubiquitylation plays some role in the activation of NF-κB downstream of STING [14].

Given that COPA is not enriched in any one immune or lung cell, its widespread expression has made it challenging to pinpoint the cell types that cause the illness. The ability of STING to target specific tissues may help us better comprehend COPA syndrome and the organs that are most impacted [12]. Considering that among monogenic type I interferonopathies, only COPA and SAVI syndrome, a mendelian disorder characterized by gain-of-function STING mutations, present with alveolar hemorrhage and ILD, this could hint at STING’s predilection toward lung involvement [21].

### 3.2. Clinical Presentations

The first report of COPA syndrome was made in 2015, following the discovery of mutations in the coatomer subunit alpha (COPA) gene in five families. These families included several members with high-titer autoantibodies, inflammatory arthritis, and childhood-onset interstitial lung disease, with nearly half of the individuals also developing renal disease [22].

The onset of clinical symptoms typically occurs in early childhood, with 3.5 years being the average age at presentation. Following the discovery of mutations in young probands, four people with symptom onset in their 50s have been described; they typically had milder disease and were found through familial sequencing [23,24,25]. A single person, whose age was accessible for the diagnosis of ILD but not the beginning of arthritis, was found through sequencing lung transplant patients and was reported as a case study [26]. Presenting symptoms are usually musculoskeletal (arthralgias to deforming arthritis) or pulmonary (tachypnea to respiratory failure). Renal failure was evident in one of the patients [22].

Years before they displayed localizing signs, a number of infants and toddlers had generic lethargy, inability to thrive, and/or anemia (caused by occult alveolar hemorrhage) [27].

Anti-nuclear antibodies (ANAs), anti-neutrophil cytoplasmic antibodies (ANCAs), rheumatoid factor (RF), and anti-cyclic citrullinated peptide antibodies (CCPs) are among the many autoantibodies present in all patients at high levels. The results for ANCA subtypes and antibodies to extractable nuclear antigens (ENAs) have varied considerably over time, both within and across individuals.

Individuals may have changes in their disease presentations over time, such as the identification of new autoantibodies and/or the involvement of new organ systems. The most common clinical symptom of COPA is arthritis, which affects 95% of patients. Four out of the twenty-one patients in a cohort by Watkin et al. had renal symptoms, such as immune complex glomerulonephritis [22]. It has also been discovered that Th1 cells that secrete IFN-γ are downregulated, whilst Th17-priming cytokines are upregulated in relation to other pro-inflammatory cytokines including IL-1β and IL-6 [22]. Th cells’ phenotypes significantly changed toward the Th17 phenotype, an effector population of T-cells engaged in the autoimmune process, as shown by Watkin et al. [22,28,29]. Psoriasis or inflammatory bowel disease (IBD) has not yet been documented in COPA patients, despite an increase in Th17 cells.

Given the recent discovery of COPA syndrome and the lack of experimental validation for the majority of the mutations reported in the literature, it is difficult to estimate the true incidence of the condition. However, 79 patients have been reported, suggesting that it is quite uncommon. There is no preference for one gender over another among patients with core COPA syndrome characteristics (39 males and 40 females).

### 3.3. Pulmonary Pathology

Pulmonary involvement is one of the more distinctive features of COPA syndrome, particularly in monogenic type I interferonopathies when it is limited to COPA syndrome and SAVI. On the other hand, pulmonary bleeding can occur in AAV and, less frequently SLE, whereas ILD can be an extra-articular manifestation of RA. There have also been reports of presentation with combined pulmonary–renal syndrome, which is a sign of AAV [30,31]. Almost all reports, involving 59 persons for whom clinical details are available, show pulmonary involvement in 98% of cases [5]. The only person who was unaffected was 9 years old at the time of the report, and earlier research had shown that there might be a 20-year delay between the onset of arthritis and ILD [32]. The first lung symptom, known as alveolar hemorrhage (AH), can appear as early as infancy [24]. Since hemoptysis is uncommon in young children, who are frequently diagnosed with pulmonary infections in the context of respiratory symptoms and patchy ground glass opacities (GGOs) on imaging, AH detection is frequently delayed [33].

Patients with COPA syndrome also frequently have a cough, tachypnoea, shortness of breath, wheezing, and/or exercise intolerance; these symptoms are linked to occult AH or indications of ILD on imaging. In COPA syndrome [30], ILD gradually develops over time, possibly as a result of ongoing bleeding and inflammation as well as an as-yet-undiscovered direct lung injury caused by COPA mutations. It is crucial to understand that, even when evident on imaging, ILD in young infants can be clinically silent. [34] Cystic alterations are one of the relatively unique imaging characteristics of COPA syndrome; other clinical entities can also exhibit GGOs, septal thickening, centrilobular nodules, lymphadenopathy (LAD), and fibrosis [30,35]. Although it was observed in less than 20% of patients, reports of crazy paving have also been made [35].

The characteristic pattern of fibrosis associated with usual interstitial pneumonia (UIP) has only been documented in one case, as described in a conference abstract. This case involved an atypical woman in her 50s who had lung disease classified as RA-ILD, and whose COPA mutation was discovered through sequencing lung transplant recipients [26]. The results of pulmonary function testing (PFT) in patients with COPA syndrome vary; they are typically mixed obstruction and restriction, followed by restriction, and isolated obstruction only in rare cases [30]. The results of the lung’s carbon monoxide (DLCO) diffusion capacity are rarely published, and they are nearly always lower (93% of 15 cases). Improvement in GGOs, nodules, and LAD is linked to immune suppression; however, cystic alterations and fibrosis are not [30,35]. Given that lung illness, the primary cause of death in COPA syndrome, can advance subclinically, this has significant clinical ramifications [30,36,37]. This progression has been reported in individuals using Janus kinase (JAK) inhibitors, as well as in patients on various combinations of immune suppressive drugs [6].

Apart from the adult-onset individual indicated above who was reported to have UIP on histology and imaging in a conference abstract, fibrosis patterns were in a non-usual interstitial pneumonitis pattern (histologic NSIP) [26]. For each patient, the histologic and radiographic patterns are usually consistent [30]. Additional neuroendocrine cell abnormalities were found on biopsy in three individuals: diffuse neuroendocrine cell hyperplasia in two and neuroendocrine hyperplasia and a carcinoid tumor in a 56-year-old man [4,25].

A number of COPA syndrome patients have advanced to bilateral lung transplantation (BLTx). There have been nine recorded transplant surgeries, several with short follow-up times. Patient outcomes range from a typical post-transplant course [4,38] to death; among them are three patients who died in the first year following their transplant [24,36] and one who died later in her post-transplant journey [26]. Two of these early fatalities occurred from related Japanese individuals whose thorough post-transplant courses have been made public [24,39]. Although survival rates vary greatly amongst lung transplant institutions, the 1-year survival rate in the US is approximately 85% [40], which is greater than the 67% observed in these COPA syndrome patients. The COPA syndrome cohort included two post-transplant deaths in patients who were bridged to transplant on long-term mechanical ventilation and/or extracorporeal membrane oxygenation (ECMO). Of these, one patient required increased immune suppression due to sensitization and the presence of a positive antibody crossmatch [36]. A small child with a serious mycobacterial infection prior to transplantation was the third early fatality [24]. As a result, it is unclear if individuals who receive transplants for COPA syndrome will fare worse than those who receive them for other reasons. The results in COPA syndrome are better than in SAVI, where just one of the five BLTx recipients survived the first year following transplantation, despite the sample size clearly being a limitation [39].

While immunological dysregulation from COPA syndrome may have hastened CLAD and/or increased the susceptibility or severity of post-transplant infections, there is no conclusive evidence yet of COPA syndrome recurrence following transplantation. Further data regarding the clinical courses following transplantation are required, including the assessment of interferon signatures and maybe JAK inhibitor trials. Further investigation is required to ascertain if combined lung and bone marrow transplants should be taken into consideration, as well as to what degree ILD in COPA syndrome is lung intrinsic, driven by hematopoietic cells, or both.

### 3.4. Arthritis

In COPA syndrome, joint symptoms are highly common and have been observed to occur prior to, following, or in addition to pulmonary disease. Moreover, 83% of the 46 patients for whom clinical data were available experienced joint symptoms, which included symmetric polyarthritis, deforming arthritis, arthralgias, and non-erosive and erosive arthritis. Arthritis sufferers can have problems with a variety of joints, such as the spine, proximal interphalangeal joints, metacarpophalangeal joints, wrists, ankles, knees, and hips. In 88% of the 17 individuals for whom a kind of arthritis was described, non-erosive arthritis was almost always present; however, it can be extremely deformative [41]. Thankfully, there are several forms of immune suppression that can be used to effectively manage joint disease symptoms and debility. Erosive illness was only documented in two patients [33,35], one of whom developed the condition from non-erosive arthritis when he was no longer receiving treatment and was lost to follow-up for eight years [33]. Abarticular involvement, in particular, tenosynovitis in the carpus, has also been reported in one patient [42].

### 3.5. Renal Pathology

Although uncommon, renal illness has been documented as the main presenting sign of COPA syndrome. A known family mutation raises the possibility of COPA syndrome. Although asymptomatic, the patient did have lung involvement, as evidenced by alveolar bleeding on BAL [19,23]. Compared to other primary COPA syndrome manifestations, published information on renal disease is more scarce and frequently restricted to biopsy results and the presence or absence. However, there are cases of severe nephropathy; the patient mentioned above and another needed kidney transplants [22,23]. The literature is not entirely clear as to whether kidney biopsies were performed in the context of increased autoantibodies in order to obtain a tissue diagnosis or to further assess aberrant laboratory tests. Within these bounds, renal involvement is present in 42% of 45 patients, or 24% of the total patient population [5].

Three patients with crescentic glomerulonephritis had positive immunofluorescence (one with positive IgM, IgG, IgA, C3, and C1q deposits, one with modest positive mesangial IgM, IgG, and C3, and another with positive IgG and C3 deposits) while four patients had negative immunological fluorescence [22,23,31,35,42,43]. Six patients exhibited focal mesangial hypercellularity with C3 and C4 staining, seven had IgA nephropathy, and eight had membranous glomerulonephritis after having an asymptomatic proteinuria biopsied as a possible kidney donor [4,22,23]. A partial nephrectomy was performed on a patient who had normal kidney function due to a nodule found on imaging. The results revealed clear cell carcinoma in the background of normal kidney tissue, which may or may not be related to COPA syndrome [25].

A 16-year-old patient presenting with stage IV renal disease and crescentic glomerulonephritis on renal biopsy concordant with AAV ended up necessitating renal transplantation after multiple immunosuppressive therapies. No follow-up data were included in the report [42].

### 3.6. Other Clinical Manifestations

A few other clinical observations have been documented in one or two COPA syndrome cases. Paroxysmal exertional dyskinesia, neuromyelitis optica [25,44], and disruptive behavior disorder are examples of neurologic involvement [45]. Hepatic cysts with cytolytic hepatitis [19], constant elevated transaminases [46], and severe gastroesophageal reflux requiring surgery are examples of gastrointestinal involvement [38,47]. Leukocytoclastic vasculitis [22], chilblain lupus [45], polymorphic rash [37], eczema [48], and vitiligo [19] are among the cutaneous symptoms. Thyroiditis [22], bacterial and viral infections, and macrophage activation syndrome are examples of immune and infectious symptoms [24,25]. There have also been reports of cardiac hypertrophy [19]. Constitutional symptoms such as fatigue and asthenia have also been reported [42]. It is uncertain to what degree these are signs of COPA syndrome [5].

### 3.7. Laboratory Investigations

The erythrocyte sedimentation rate (ESR), which is rarely higher than 90 mm/h, is mildly to moderately raised in 95% of the 23 patients with COPA syndrome, and many of these patients have elevated inflammatory markers. In contrast, in 91% of the 23 individuals whose laboratory data were reported, the C-reactive protein (CRP) was generally normal to slightly raised, at less than 15 mg/L. According to previous reports, an individual’s ESR and CRP can differ, with increases somewhat corresponding to disease activity. For example, a patient may experience improvement in their ESR one year after their severe arthritis resolved, but their CRP may not return to normal [47].

In six out of six cases, the ultra-sensitive digital ELISA test revealed increased IFNa levels, typically ranging from 150 to 1700 fg/mL [19]. Interleukins (IL) were raised in a number of individuals, including soluble IL-2 receptor A in one patient, IL-1b in one patient, and IL-6 in three patients. Research-based testing also revealed that 100% of the 13 patients tested had high interferon signatures [19,24,32,34,42,48], one of whom had resolution with baricitinib therapy [24] and another presented a significant decrease with ruxolitinib therapy [42]. Increased IFNa2 was noted in another case [32].

### 3.8. Autoantibodies

All COPA syndrome patients showed high ANA, ANCA, and/or RF/CCP, with the exception of one. Of the 59 individuals whose autoantibody results were specifically reported, 72% were ANA positive, a little higher than the 67% to 70% previously reported [6]. When titers were reported, they were nearly always 1:160 or higher, and frequently 1:640 or higher. The reported staining patterns varied greatly, appearing scattered, speckled, or homogenous. While dsDNA, Smith, and RNP antibodies were detected in at least one case, ENAs were reported less frequently. As evidenced by a patient in whom serial testing over a 14-month period revealed that each of these antibodies was only found once, they can change over time and depend on the severity of the condition [49]. On the other hand, it has been noted that ANA positivity is persistent.

Six people had PR3, including some with fluctuating positive and low titers, while thirteen people had MPO, which was often detected at high titers [42,43,49]. With disease activity, MPO and PR3 may change, while ANCA stays positive [42,43,49]. Similar to the 43% to 61% previously reported, 66% of reported patients tested positive for CCP and/or RF [6]. Titers were typically significantly raised to more than 100 IU/mL for RF and more than 50 U/mL for CCP when reported, with several values beyond the limit of detection. In conclusion, extractable nuclear antigens are uncommon, and a high-titer ANA is a characteristic of COPA syndrome. Analyses conducted serially showed that the ANA remained positive, but the ENAs varied significantly and did not predict disease activity. Compared to PR3, ANCA is more frequently positive and can be observed alone or in conjunction with MPO. High titers are also typical for RF and CCP. Multiple autoantibodies, irrespective of phenotype, are a very distinctive hallmark of COPA syndrome; several individuals have been found to have high titer levels of ANA, ANCA, and RF/CCP.

### 3.9. Diagnostic Red Flags and When to Test

Because initial symptoms of COPA syndrome can resemble those of other autoimmune disorders such as rheumatoid arthritis, ANCA vasculitis, and systemic lupus erythematosus, early identification can be difficult. This presents a real challenge considering SAVI syndrome, a STING-related interferonopathy that also presents with ILD and DAH and is characterized by poor life expectancy in the first two decades of life due to progressing lung disease, making early diagnosis of COPA syndrome a necessity as the two diseases might have similar prognoses [3].

The type I IFN pathway should be assessed, and regular laboratory tests, if not already performed, should be suggested if typical clinical indications of COPA syndrome are present. Since lung disease can be the only manifestation of COPA syndrome, this diagnosis should be suspected in cases of unexplained DAH and ILD. It is worth noting that some patients can present with occult alveolar hemorrhage.

We summarize the red flags that should prompt testing for COPA syndrome in Table 1.

Routine laboratory tests are crucial for gathering evidence of autoimmunity and inflammation, as well as for identifying the affected organs, even if they are not specific to COPA syndrome. Notably, an inflammatory condition exhibits irregularities and contradictory ESR and CRP readings. DAH can manifest with few or no symptoms in patients with COPA who have inflammatory arthritis and/or kidney disease. It can be identified by non-specific biological data that mimic hemolytic anemia, which is characterized by low hemoglobin levels combined with high reticulocyte counts and low blood-iron levels. Routine laboratory tests will also be used as reference values for further assessments of the severity of the disease and the effectiveness of treatment. Non-exhaustively, these include immunophenotyping (Th17 cells), autoantibodies (ANA, ANCA, anti-CCP, and rheumatoid factor), inflammatory markers (hemogram, ESR, and CRP), signs of chronic bleeding (reticulocytes and iron), and organ-specific markers (TSH, proteinuria, urine creatine, serum creatine, and liver enzymes) [6].

A high titer of autoantibodies in a patient with pulmonary disease and refractory pulmonary symptoms in patients already diagnosed with RA, ANCA vasculitis, or SLE should present diagnostic red flags and guide the clinician toward testing for COPA syndrome.

A further argument in favor of genetic testing is the discovery of an IFN pathway induction confirmed at least twice, in the absence of recent viral infection and in the presence of suggestive characteristics of COPA syndrome [50].

### 3.10. Treatment

Since there is currently no specific treatment for COPA syndrome, immunosuppressives derived from the treatment of other immunological illnesses that share characteristics are used to treat its manifestations.

Exacerbations and life-threatening alveolar hemorrhage are generally controlled with pulse-dose IV steroids, intravenous immunoglobulin (IVIG), pulse-dose cyclophosphamide, or rituximab. It is worth noting that three reported exacerbations treated with cyclophosphamide [43,51,52] required follow-up treatment with rituximab for disease control. One of the cases mentioned ended with patient death after an infectious complication during rituximab treatment [51].

Maintenance therapy has been tried with oral corticoids, varying classes of synthetic immunosuppressives, and biological therapies. We attempt to summarize the reported maintenance therapy strategies used in COPA patients that mentioned said strategies and included follow-up data for at least one type of involvement in Table 2. Please note that clinical response to these therapies has been interpreted as reported by the authors, as the results are extremely heterogeneous in terms of both frequency and parameters of monitoring, and with disease understanding being very low currently, it is difficult to objectively assess clinical response.

Patwardhan et al. [33] reported two cases of COPA syndrome, a father and a son, both with juvenile onset. The son continued to have respiratory symptoms and pulmonary hemorrhages despite corticoids and methotrexate (MTX) combination therapy and then azathioprine (AZA), which warranted switching to mycophenolate mofetil (MMF) with positive evolution and no relapse of lung disease. The father presented with severe arthritis after 8 years of having been lost to follow-up. Adalimumab therapy was initiated after etanercept, and MTX therapy was not able to control the disease. It is unclear from the report whether adalimumab was efficient in controlling pulmonary disease [33].

One case that was initially diagnosed as JIA at the age of 4 and treated with MTX and corticoids with poor disease control, eventually leading to macrophage activation syndrome, underwent pulse-dose cyclophosphamide treatment follow-up with rituximab and continued with mycophenolate and hydroxychloroquine and prednisone as maintenance therapy. The author reported the stabilization of lung involvement [52].

One case was treated with abatacept for joint involvement, with poor management of arthritis disease activity—with better results after switching to sarilumab [54].

One 12-year-old boy with COPA syndrome was reported by Noorelahi et al. [56]. There was a significant restrictive lung defect first, followed by polyarticular arthritis. Disease activity was effectively controlled under adalimumab, methotrexate, and naproxen [56].

Th17-focused therapy seems to show some promise as there is evidence of two patients, presenting with arthritis, ILD, and follicular bronchiolitis, having significant improvement of pulmonary involvement after treatment with mTOR inhibitor sirolimus. The patients treated with sirolimus showed decreased serum IL-6 and type I IFN signature, but they also required sirolimus serum titer monitoring to avoid toxicity [48].

Krutzke et al. presented a case with ILD and arthritis treated with combination therapy of baricitinib and methotrexate, with significant improvement in joint involvement and stabilization of pulmonary function [47].

JAK-inhibitor therapy seems to be the most promising therapeutic choice at this moment as there are multiple cases of successful treatment with baricitinib, one with tofactinib, and two with ruxolitinib, efficient in treating both joint and lung manifestations, albeit with varying degrees of success. One of the patients treated with ruxolitinib required a switch to baricitinib and an associated IL-1 blocker after a relapse of alveolar hemorrhage and ILD progression on a CT scan [6,27,34,47]. It is worth noting that the patient treated with tofacitinib as a maintenance therapy also had an induction with cyclophosphamide and rituximab, and there might be an overlap in clinical response between the therapies.

Cartagena et al. [42] presented the case of a 16-year-old boy presenting with stage IV renal failure, tenosynovitis, and cystic lung disease. The patient was initially diagnosed with AAV after a compatible renal biopsy and positive autoantibodies and underwent treatment with pulse methylprednisolone, cyclophosphamide, four doses of rituximab every 6 months, and seven sessions of plasmapheresis. After the COPA diagnosis and a high interferon signature test, the patient started maintenance therapy with ruxolitinib and underwent renal transplantation. In terms of pulmonary involvement, the authors report a slight improvement in functional pulmonary tests and the absence of ILD progression [42].

The argument for JAK inhibitor therapy being a prime choice for COPA syndrome treatment is also supported by their usage in treating other interferonopathies such as CANDLE syndrome and SAVI syndrome, which is closely related to COPA in terms of etiology and manifestations. However, the quality of the evidence is low, and further clinical trials are needed to assess safety and efficacy [57,58].

It is worth mentioning that children might require higher doses of JAK inhibitors compared to adults, as outlined by Kim et al. in their paper concerning JAK inhibitor treatment of mendelian interferonopathies [59].

It should be noted that, currently, JAK inhibitor medication can only be used off-label in pediatric patients as their safety profile in said demographic has not been thoroughly tested. Patients suitable for treatment with JAK inhibitors may be identified by an increased interferon score. In a case series of seven children with interferon-mediated inflammatory diseases treated with off-label baricitinib, clinical improvement was recorded in all cases, and two of the children presented adverse drug reactions, one of which was considered serious [55].

When it becomes accessible, specific medications such as STING inhibitors—which target both the interferon-dependent and interferon-independent functions of STING—may be a more promising choice. They are now in the late stages of clinical research [60].

When it comes to ILD, progressive fibrosing phenotypes could benefit from treatment with tyrosine-kinase inhibitors such as nintedanib, a proven antifibrotic agent [61]. Another promising treatment for ILD seems to be pirfenidone, which slows the rate of decline of FVC in patients with RA-related ILD according to TRAIL1—a randomized, double-blind, placebo-controlled phase 2 trial. However, as the study was terminated early due to the COVID-19 pandemic, its results should be interpreted with caution [62]. As of yet, there have been no reports of antifibrotic therapy being used in COPA patients.

## 4. Discussions

The hereditary immune dysregulation condition known as COPA syndrome is relatively recent and is characterized by high autoantibody titers, lung disease, and arthritis. Genetic sequencing COPA confirms the diagnosis. When a mutation is found, familial testing for healthy at-risk persons should be taken into consideration because the non-penetrance rate is at least 15%. To understand the causes of illness non-penetrance, more research is required [5].

Early diagnosis can be difficult due to initial presentations mimicking other autoimmune diseases such as rheumatoid arthritis, ANCA vasculitis, and systemic lupus erythematosus—arthritis/kidney/lung involvement associated with ANA/ANCA/anti-CCP antibodies; while respiratory symptoms are usually indistinguishable from pneumonia—cough, dyspnea, tachypnoea. Thus, a patient with an early-onset disease presenting with lung involvement and high-titer autoantibodies or a patient initially diagnosed with the autoimmune diseases mentioned above presenting with refractory lung disease should present diagnostic red flags, especially in the case of a positive family history of lung disease.

The mainstay of care is immune suppression. Exacerbations usually require induction therapy with pulse-dose corticoids, cyclophosphamide, or rituximab [6]. The efficacy of maintenance therapy is difficult to ascertain as there are not enough long-term follow-up data currently reported. Current evidence points toward JAK inhibitor therapy as a primary choice for immunosuppression, also supported by its efficacy in treating other interferonopathies [58,59]. Given that the disease mechanisms behind COPA syndrome skew T-cells toward the Th17 phenotype, mTOR inhibitors could be beneficial, with current evidence being scarce but promising [48]. Specific STING inhibitors could present a future option if clinical trials prove successful [60].

While a swathe of immunosuppressive therapies has shown success in managing arthritis, the stabilization of lung disease is tricky to ascertain as it requires thorough and long-term monitoring, with present data being inconclusive. This presents a real challenge as SAVI syndrome, another STING-related interferonopathy presenting with ILD and DAH, is characterized by poor life expectancy in the first two decades of life due to progressing lung disease, and COPA syndrome might follow a similar pattern [3]. Antifibrotic therapy, although an untested therapy in this syndrome, might prove a useful tool for slowing down ILD progression.

Even though there is evidence of interferon scores decreasing under JAK-inhibitor treatment, it is unclear whether the interferon score assay could be used as marker when it comes to monitoring disease activity.

Patient registries would be beneficial for studying COPA syndrome further as they would assist in explaining rates of illness prevalence and penetrance, expanding the understanding of disease symptoms, and directing treatment decisions. In the absence of large-scale studies, detailed updates on previously reported cases could also provide useful information for disease management.

## Figures and Tables

**Table 1:** Red flags that should guide genetic testing in COPA syndrome.

Diagnostic Red Flags		
Clinical	Paraclinical	Imaging
Early on-set age	Histopathology: follicular bronchitis, histologic NSIP pattern	Diffuse alveolar hemorrhaging
Cough, dyspnea, tachypnoea, hemoptysis	Positive ANA/ANCA/anti-CCP/RF serology	ILD—cysts, fibrosis
Arthritis and/or renal involvement	High interferon assay score	
+ refractory respiratory symptoms or positive family history of lung disease		

**Table 2:** Maintenance therapy efficacy on joint/lung/renal progression as reported by the respective authors.

Patient No.	Author	Maintenance Therapy	Joint Prog	Lung Prog	Renal Prog
1	Oliveira et al. [53]	AZA + HCQ			
2	Guan et al. [48]	Corticoids + MTX			
		Sirolimus			
3	Guan et al. [48]	Corticoids			
		Sirolimus			
4	Krutzke et al. [47]	ETN + MTX			
		Baricitinib + MTX			
5	Brennan et al. [52]	Corticoids + MTX			
		Corticoids + MMF + HCQ + ATB *			
6	Patwardhan et al. [33]	Corticoids + MTX			
		AZA			
		MMF			
7	Patwardhan et al. [33]	ETN + MTX			
		ADA + MTX			
8	Basile et al. [34]	Baricitinib			
9	Frémond et al. [27]	Ruxolitinib			
		Baricitinib + IL-1b			
10	Nikolic et al. [54]	Abatacept			
		Sarilumab			
11	Zheng et al. [43]	Tofacitinib *			
12	Pin et al. [55]	Corticoids + MTX + MMF			
		Baricitinib + MMF			
13	Noorelahi et al. [56]	ADA + MTX			
14	Cartagena et al. [42]	Ruxolitinib *			
	Responsive				
	Relapse/partially responsive				
	Non-responsive				
	Not reported/Not applicable				

MTX = Methotrexate; MMF = Mycophenolate; AZA = Azathioprine, HCQ = Hydroxychloroquine; ETN = Etanercept; ADA = Adalimumab; ATB = prophylactic antibiotics, IL-1b = interleukin 1 blocker. * Patients received induction therapy with Cyclophosphamide and Rituximab.

## Data Availability

No new data were created or analyzed in this study.
